# Effect of Laser Beam Profile on Thermal Transfer, Fluid Flow and Solidification Parameters during Laser-Based Directed Energy Deposition of Inconel 718

**DOI:** 10.3390/ma16124221

**Published:** 2023-06-07

**Authors:** Bo Chen, Yanhua Bian, Zhiyong Li, Binxin Dong, Shaoxia Li, Chongxin Tian, Xiuli He, Gang Yu

**Affiliations:** 1 Institute of Mechanics, Chinese Academy of Sciences, Beijing 100190, China; chenbo@imech.ac.cn (B.C.); bianyanhua@imech.ac.cn (Y.B.); lizhiyong@imech.ac.cn (Z.L.); dongbinxin@imech.ac.cn (B.D.); lisx@imech.ac.cn (S.L.); tianchongxin@imech.ac.cn (C.T.); 2 Center of Materials Science and Optoelectronics Engineering, University of Chinese Academy of Sciences, Beijing 100190, China; 3 School of Engineering Science, University of Chinese Academy of Sciences, Beijing 100190, China; 4 Guangdong Aerospace Research Academy, Guangzhou 511458, China

**Keywords:** laser profile, thermal behavior, fluid flow, dimensionless number, cooling rate

## Abstract

The profile of the laser beam plays a significant role in determining the heat input on the deposition surface, further affecting the molten pool dynamics during laser-based directed energy deposition. The evolution of molten pool under two types of laser beam, super-Gaussian beam (SGB) and Gaussian beam (GB), was simulated using a three-dimensional numerical model. Two basic physical processes, the laser–powder interaction and the molten pool dynamics, were considered in the model. The deposition surface of the molten pool was calculated using the Arbitrary Lagrangian Eulerian moving mesh approach. Several dimensionless numbers were used to explain the underlying physical phenomena under different laser beams. Moreover, the solidification parameters were calculated using the thermal history at the solidification front. It is found that the peak temperature and liquid velocity in the molten pool under the SGB case were lower compared with those for the GB case. Dimensionless numbers analysis indicated that the fluid flow played a more pronounced role in heat transfer compared to conduction, especially in the GB case. The cooling rate was higher for the SGB case, indicating that the grain size could be finer compared with that for the GB case. Finally, the reliability of the numerical simulation was verified by comparing the computed and experimental clad geometry. The work provides a theoretical basis for understanding the thermal behavior and solidification characteristics under different laser input profile during directed energy deposition.

## 1. Introduction

Additive manufacturing (AM) technology is increasingly becoming a popular manufacturing technique for producing parts from various metals and alloys, such as stainless steels, Ni-based superalloys, Al-alloys, etc. [1,2,3,4]. Among AM techniques, laser-based directed energy deposition (L-DED) is considered as a promising manufacturing method due to its unique merits, such as little heat affected zone, low dilution, and fine grain size [5,6]. It is widely used in material manufacturing, such as damaged parts repairing [7], metallic coating [8], etc. During the process of L-DED, the metallic substrate absorbs the energy of the laser beam and forms a small molten pool where powder particles are transported through a powder delivery system. As the laser moves along together with the feeding system, the solidified clad layer forms. Complex physical phenomena, such as laser–powder interaction, mass addition, fluid flow and temperature changes, are usually coupled in the process [9]. Due to the highly transient process of rapid heating and cooling, experimental measurement of those complex physical phenomena is difficult. As a result, an accurate numerical model based on the physics, validated using the experimental data, can help us understand the underlying theory of molten pool dynamics during L-DED processing.

A substantial amount of numerical model has been proposed based on reasonable simplifications because of the complex physical phenomena involved in the L-DED process. Dortkasli et al. [10] developed an efficient finite element model for thermal process to predict the characteristics of the clad layer in the L-DED process. However, the fluid flow of the liquid metal was not taken into account in the simulation. Sun et al. [11] established a 3D numerical model to study the mass and thermal transport in a high deposition rate laser cladding process. In this model, the interaction between powder particles and the molten pool surface was considered. Gan et al. [12] proposed a self-consistent model, which incorporated the fluid dynamics and multicomponent mass transfer. In this model, mass transfer and fluid flow were coupled successfully and the solidification parameters were also analyzed. Li et al. [13] developed a multi-physics model for DED process of functionally gradient materials (FGMs) fabrication. This model can predict the solute distribution and geometry of the manufactured FGMs. However, none of the above models took the interaction between laser beam and powder flow into account directly.

The attenuation of laser beam by powder particles and the heat absorption of powder particles are two key factors which dominate the energy input and further influence the fluid flow throughout the L-DED process. Bayat et al. [14] established a multi-physics numerical model to investigate the molten pool variation affected by different particle velocities. In this model, the impingement velocity and temperature rise of powder particles were taken into account. However, the model did not contain the influence of powder streams on the laser intensity. Song et al. [15] created a numerical model for laser metal deposition process and used various process parameters to compare with experimental results. In this model, the attenuation of laser was calculated using a semi-empirical model. However, the powder temperature distribution on the deposition surface was not involved in this model. Wang et al. [16] developed a 3D numerical model for L-DED, considering thermal-fluid transport and laser–powder interaction. Although the effect of laser–powder interaction was considered, thermal convection and radiation of the powders were ignored. Moreover, no detailed consequence of the attenuated laser intensity and powder temperature rise were reported. Wu et al. [17] fabricated a 2D numerical model to analyze the thermal-fluid transport inside the molten pool. In this model, whole-phase laser–powder coupling and material deposition were comprehensively considered. Yet, the analysis of the L-DED process is limited by the model dimension.

The process parameters determine the solidification conditions and further the quality of the molten pool. Intensity profile of laser beam is a key parameter which plays a significant role in the laser material processing. Gaussian, super-Gaussian and flat top profile are three typical distribution of laser beam intensity. For a focused fiber laser beam, the beam profile transforms from flat-top to Gaussian from the focal plane to the far field [18]. The super-Gaussian function is used to describe the spatial profile of laser beam from Gaussian profile to a flat-top profile. Several researches [18,19,20,21,22,23] have been proposed to explore the effect of laser intensity on the laser-induced molten pool. Han et al. [19] used different types of laser beam modes to study their influence on the molten pool and concluded that the molten pool under Gaussian beam had the greatest depth. Ayoola et al. [20] claimed that a Gaussian laser beam could result in deeper weld pool compared with that for a laser beam with top-hat profile. Kaplan [18] indicated that a top-hat beam and Gaussian beam would cause different, steep keyhole shapes during deep penetration laser welding. Huang et al. [21] found that a top-hat laser beam was more suitable for manufacturing a denser prototype compared with a Gaussian beam in laser powder bed fusion (LPBF). This study showed that a lower aspect ratio and less keyhole porosities for the clad were observed in the case using a top-hat laser beam. Yuan et al. [22] investigated the thermal-fluid transport inside the molten pool under the laser beams with flat-top and Gaussian profile in LPBF. The results showed that these two laser beams had significantly different effect on the clad appearance. Moreover, the flat-top laser showed great potential for controlling the directed growth of grains. Wu et al. [23] compared the different phenomenon of thermal behavior and fluid flow inside the molten pool under Gaussian and super-Gaussian beam via a two-dimension model for the L-DED process. It was found that the beam with super-Gaussian profile had less influence on the depth of the molten pool and had better stability than that with the Gaussian distribution.

As seen from the above discussions, powder addition and laser intensity are influential on mass input and heat flux input applied to the deposited surface, which would largely affect the thermal-fluid transport. The research on mutual coupling influence of the laser energy input and powder addition behavior is essential for a comprehensive understanding of the L-DED process. There have been some studies [14,15,16] on the mutual coupling effect of powder addition behavior and the laser intensity input with Gaussian distribution. However, there has been few researches on the laser–powder interaction and corresponding heat transport of powders under the laser intensity input with super-Gaussian distribution. Therefore, in this paper, an improved thermal-fluid model including a laser–powder interaction model and a metal deposition model was established to explore the thermal-fluid transport and solidification characteristics under two types of laser beams (Gaussian and super-Gaussian) during single-track L-DED process of Inconel 718. The moving mesh method [12,15] was used to capture liquid/gas interface dynamically and the apparent heat capacity method [16,17] was used to track the solid/liquid interface. Subsequently, several dimensionless numbers were analyzed to explain the molten pool evolution during the L-DED process using the calculated results. Furthermore, the solidification parameters which are obtained from thermal history were used to reveal the solidification patterns and grain morphology of the molten pool. Finally, the reliability of the model was verified by comparing the morphology of the molten pool from calculations and experiments.

## 2. Experimental Procedure

**Figure 1:**
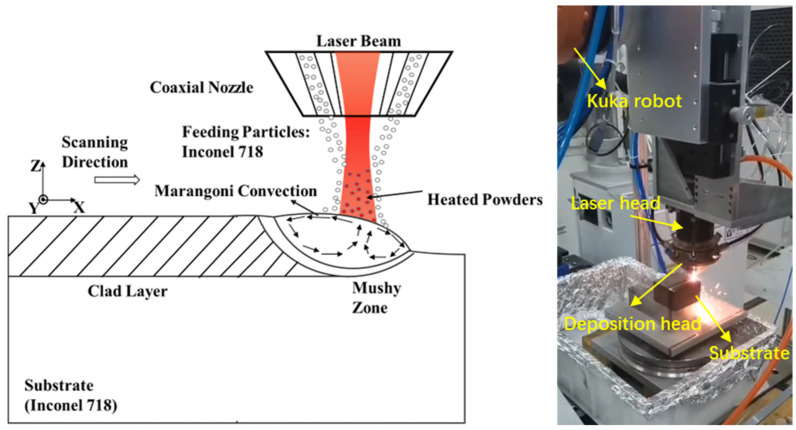
Schematic diagram and experimental setup for coaxial L-DED process.

To validate the consequence of the 3D transient thermal-fluid model, single-track deposition experiments were carried out on a fiber laser system equipped with a coaxial powder feeding nozzle. The schematic diagram and experimental setup for coaxial L-DED process are plotted in Figure 1.

**Figure 2:**
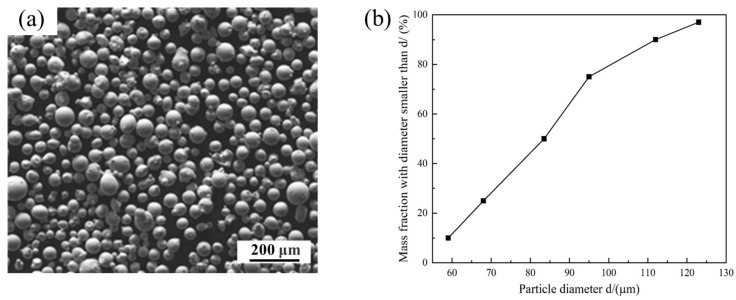
(**a**) SEM image and (**b**) Cumulative distribution of Inconel 718 powder particles.

The substrate supplied by Shanghai Linzhi Metal Materials Co., Ltd. (Shanghai, China) and powder supplied by Jiangsu Vilory Advanced Materials Technology Co., Ltd. (Xuzhou, China) were both Inconel 718. The substrate with the dimensions of 100 mm × 40 mm × 40 mm was sanded using 40 #, 180 # and 400 # silicon carbide sandpaper and washed with ethanol before experiments. Powder particle diameter is in the range of 59–123 μm, as shown in Figure 2a, and the cumulative distribution is plotted in Figure 2b.

The wavelength of the fiber laser (YLS-10000-CUT, IPG Photonics Corporation, Oxford, MA, USA) used in the experiments was 1070 nm. The spot radius of laser beam was set as 1.6 mm. Both the carrier gas and the protective gas were argon with a purity of 99%. A continuous coaxial powder feeding nozzle with two channels for shielding gas and an annular wedge-shaped channel for powder and carrying gas was employed to provide a protective atmosphere and the added powder material. The detailed structure of the continuous coaxial powder feeding nozzle can be checked in our previously published paper [24]. The movement of the laser cladding system was implemented and controlled by a six-axis KUKA robot. More parameters used for experiments are presented in detail in Table 1.

## 3. Mathematical Model

As stated in the Introduction section, L-DED of alloys is a very complex process. In order to establish a model for the whole L-DED process, some reasonable assumptions are as follows [9,25,26]:

(1) Liquid metal flow inside the molten pool is considered as Newtonian, laminar and incompressible.
(2) The vaporization is ignored because the calculated peak temperature of the system is lower than the boiling point of the material.
(3) The mushy zone whose temperature is between the solidus and liquidus is described using a porous medium with isotropic permeability.
(4) The impact of shielding gas is not taken into consideration because of its minimal pressure.
(5) The powder instantly melts when it gets in touch with the deposition surface and is treated as a continuous and uniform phase.

### 3.1. Governing Equations

According to the above assumptions, the conservation equation of mass is:

(1) $$\frac{\partial \rho }{\partial t}+\nabla \cdot (\rho u)=0$$

where, ***u*** and *ρ* denote the velocity and density of metal fluid, respectively.

The conservation equation of momentum is:

(2) $$\frac{\partial (\rho u)}{\partial t}+\nabla (\rho \mathrm{uu})=-\nabla p+\nabla (\mu (\nabla u+\nabla u^{T}))+F_{b}+F_{m}$$

where, *p* is the pressure, and μ is the dynamic viscosity. The second term on the right hand of Equation (2) is related to viscosity shear stress. $F_{b}$ is used to describe the force of buoyancy using Boussinesq approximation [27], and its formula is given as,

(3) $$F_{b}=(1-\beta (T-T_{ref}))\rho g$$

where, ***g*** is the gravity acceleration, β is the coefficient of volumetric expansion, and *T_ref_* is the reference temperature. The last term on the right hand of Equation (2) represents the damping force and it is given as [28],

(4) $$F_{m}=-A_{mushy}\frac{{\left(1-f_{l}\right)}^{2}}{f_{l}{}^{3}+\varepsilon }u$$

where, *A_mushy_* is a huge constant (10^4^ in this paper) related to the damping forces, and *ε* is a positive constant (10^−3^ in this paper) to avoid the division by zero. *f_l_* represents the fraction of liquid phase in the mushy zone, which could be given as:

(5) $$f_{l}=\left\{\begin{array}{cc} 0 & \left(0<T<T_{s}\right)\\ \frac{T-T_{s}}{T_{l}-T_{s}} & \left(T_{s}<T<T_{l}\right)\\ 1 & T>T_{l} \end{array}\right.$$

where, *T* is the local temperature of the system. *T_s_* and *T_l_* denote the solidus and liquidus temperature of the liquid metal, respectively.

The conservation equation of thermal energy is:

(6) $$\rho c_{p}\frac{\partial T}{\partial t}+\rho c_{p}u\cdot \nabla T=k\nabla^{2}T$$

where, *c_p_* and *k* represent the heat capacity and thermal conductivity of the metal, respectively.

### 3.2. Heat Source Model

**Figure 3:**
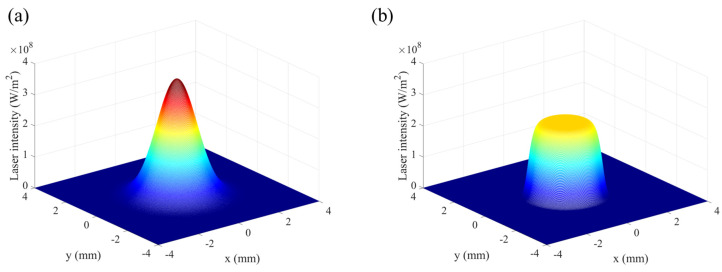
Laser intensity under different super-Gaussian order of (**a**) 1, (**b**) 5 (laser power: 1500 W; laser spot: 1.6 mm).

The laser heat source applied on the liquid/gas interface is assumed to be super-Gaussian beam (SGB) and its spatial laser intensity profile can be expressed as [29]:

(7) $$I_{SGB}(x,y)=\frac{4^{1/N}NP}{2\pi r_{l}{}^{2}\Gamma (N/2)}\exp[-2{\left(\frac{{\left(x-v_{s}t\right)}^{2}+y^{2}}{r_{l}{}^{2}}\right)}^{N/2}]$$

where, *P* is the input laser power and *N* represents the order of super-Gaussian. Γ denotes the gamma function and Γ(*N/2*) is the SGB power distribution. The beam radius is related to the axial propagation of the laser and it is given as [18]:

(8) $$r_{l}=r_{0}\sqrt{1+{\left(\frac{z-z_{0}}{z_{R}}\right)}^{2}}$$

where, *z_R_* is the Rayleigh range. While *N* = 1, there is a Gaussian profile for the energy source, which is presented in Figure 3a. According to the high-power fiber laser used in laser cladding experiments, the value of *N* is approximately 5. As shown in Figure 3b, the heat source intensity looks like a top-hat and the total energy is divided equally within the limited region.

### 3.3. Laser–Powder Interaction

For laser–powder interaction in the L-DED process, two main physical phenomena are involved: laser intensity attenuation and temperature rise of powders. The concentration distribution of the coaxial powder stream is assumed to be a Gaussian distribution [30] and it is given as:

(9) $$N(r,z)=\frac{2M_{p}}{m_{p}\pi v_{p}R_{p}{}^{2}(z)}\exp(-\frac{2\cdot r^{2}}{R_{P}{}^{2}(z)})$$

where, *M_p_* is the mass flow rate, *R_p_* represents the equivalent Gaussian radius at each cross section, *v_p_* denotes the powder velocity and *m_p_* is the mass weight of a single powder particle.

Due to the absorption and scattering of the powder stream, the attenuation of the laser beam intensity can be calculated using Beer Lambert law [31]:

(10) $$dI=-Q_{ext}\pi r_{p}{}^{2}I(r,z)N(r,z)dz$$

where, *Q_ext_* is the extinction coefficient. In this study, the average particle radius *r_p_* = 42 μm, and laser wavelength λ*_l_* = 1.07 μm. The laser wavelength is much smaller than the particle size. Thus, it is assumed that the powder particles absorb most of the attenuated energy and the extinction coefficient *Q_ext_* = 1 [31].

By integrating Equation (10) along the z-direction, the attenuated laser beam intensity *I_a_* (*r*, *z_k_*) can be calculated using the following formula:

(11) $$I_{a}(r,z_{k})=\left\{\begin{array}{cc} I_{SGB}(r,z_{0}) & k=0\\ I_{a}(r,z_{k-1})\exp(-Q_{ext}\pi r_{p}{}^{2}N(r,z_{k-1})(z_{k}-z_{k-1})) & k\geq 1 \end{array}\right.$$

where, *z*_0_ is the initial interaction position of the laser beam and powder stream.

The powder particle temperature starts to rise as it absorbs the laser energy. Moreover, as it absorbs sufficient energy, phase changes could occur before reaching the liquid/gas interface. The temperature rise for a single powder particle follows the heat balance equation [17]:

(12) $$m_{p}c_{p}\frac{dT_{p}}{dt}=\eta_{p}I_{a}(r,z)\pi r_{p}{}^{2}-h_{p}A_{p}(T_{p}-T_{\infty })-\varepsilon \sigma A_{p}(T_{p}^{4}-T_{\infty }{}^{4})-m_{p}L_{m}\frac{df}{dt}$$

where, *η_p_* denotes the laser absorptivity, *h_p_* is convective heat transfer coefficient of the powder, *A_p_* is the surface area of the particle, *T_∞_* represents the temperature of surrounding gas, and *L_m_* represents the latent heat of the particle material. The first term on the right hand of Equation (12) represents the heat absorbed by powder particles. The second and third term denote the convection and radiation between powder particles and the environment, respectively. The last term is relative to the phase change of powder particles.

### 3.4. Phase Change

After the temperature of the heated metal exceeds the liquidus, a solid–liquid mixed zone begins to appear. The thermal property of the mixed-region between solid and liquid is determined using a linear equivalent treatment, the formula is as follows:

(13) $$\rho =\theta_{s}\rho_{s}+\theta_{l}\rho_{l}$$

(14) $$k=\theta_{s}k_{s}+\theta_{l}k_{l}$$

where, *θ_i_*, *ρ_i_* and *k_i_* represent the volume fraction, density and thermal conduction of *i* phase, respectively. The phase change in the molten pool is tracked using the apparent heat capacity method and the related formula is expressed as [32]:

(15) $$c_{p}=\frac{1}{\rho }(\theta_{s}\rho_{s}c_{ps}+\theta_{l}\rho_{l}c_{pl})+L_{m}\frac{\partial \alpha_{m}}{\partial T}$$

where, $\alpha_{m}$ is a distribution function of the latent heat which can be given as:

(16) $$\alpha_{m}=\frac{1}{2}\frac{\theta_{l}\rho_{l}-\theta_{s}\rho_{s}}{\rho }$$

The thermal property of the material applied in the calculation is presented in Table 2.

### 3.5. Free Surface Tracking

The free surface is tracked explicitly using the Arbitrary Lagrange–Euler (*ALE*) method and its normal velocity can be expressed as [12]:

(17) $$V_{L/G}=u\cdot {n+v}_{a}\cdot n$$

where, ***n*** stands for the unit normal vector of the free surface and ***v_a_*** is the related moving velocity due to mass addition, which can be described as:

(18) $$v_{a}=\frac{2\eta_{c}M_{p}}{\rho_{p}\pi R_{p}{}^{2}}\exp[-2\frac{{\left(x-v_{s}t\right)}^{2}+y^{2}}{R_{p}{}^{2}}]\phi_{s}(T)e_{z}$$

where, *ϕ_s_* (*T*) is a smooth function, which is defined as:

(19) $$\phi_{s}(T)=\left\{\begin{array}{cc} 0 & T<T_{s}\\ 0.5+0.5\sin(\pi \frac{T-(T_{l}+T_{s})/2}{T_{l}-T_{s}}) & T_{s}<T<T_{l}\\ 1 & T>T_{l} \end{array}\right.$$

where, *η_c_* is the capture efficiency of the powders. The smooth function is used to ensure that the powder could only be captured by the melt metal. On the other hand, the powder has no influence on the no-melting substrate whose temperature is below the melting point.

### 3.6. Boundary Conditions

Due to powder injection, the free surface grows into a curved surface which separates the gas and liquid phases. Based on the previous assumption that as long as powders attach to the molten pool, they melt instantly and mix with the liquid metal, the free surface is treated as continuous media. Only half of the calculated domain is contained in this model because of the symmetry during the L-DED process.

#### 3.6.1. Momentum Boundary Conditions

The liquid/gas interface stress tensor can be divided into two temperature-dependent parts in normal and tangential direction, which can be given as [7]:

(20) $$F_{L/G}=\sigma \kappa n-\gamma (\nabla T-(\nabla T\cdot n)n)$$

The first force is capillary force in normal direction pointing into the liquid metal. *κ* represents the curvature of the free surface and σ denotes the surface tension, which are expressed as follows [26]:

(21) κ=∇⋅n

(22) $$\sigma =\sigma_{ref}+\gamma (T-T_{ref})$$

where, *T_ref_* and *σ_ref_* represent the reference temperature and surface tension, respectively. The second force of Equation (20) is the Marangoni force in the tangential direction which is related to variation of the surface tension. *γ* denotes the surface tension coefficient.

Due to the symmetry of the surface conditions, the fluid velocity is limited on the symmetry surface, which means that y-direction flow is forbidden in the plane, thus, the law of limited fluid flow is given as:

(23) $$u\cdot n_{sp}=0$$

where, $n_{\mathrm{sp}}$ is the normal phasor of the plane.

Accordingly, the stress boundary conditions in the symmetric plane can be expressed as:

(24) $$\{\mu [\nabla u+{\left(\nabla u\right)}^{T}]\}n_{sp}-((\{\mu [\nabla u+{\left(\nabla u\right)}^{T}]\}n_{sp})\cdot n_{sp})n_{sp}=0$$

#### 3.6.2. Thermal Boundary Conditions

The heat flux applied on the free surface can be expressed as:

(25) $$-k\frac{\partial T}{\partial n}=\eta_{l}I_{a}-q_{p}-h_{c}(T-T_{\infty })-\varepsilon \sigma (T^{4}-T_{\infty }{}^{4})$$

where, *I_a_* represents the attenuated laser power intensity which is calculated using Equation (11). *η_l_* is the laser absorptance of the substrate. *q_p_* is a source term carried by the heated powders and it can be expressed as:

(26) $$q_{p}=\left\{\begin{array}{ll} M_{p}{}^{\prime \prime }[L_{m}+c_{p,s}(T_{s}-T_{p})+c_{p,l}(T-T_{l})]\phi_{s}(T) & T_{p}<T_{s}\\ M_{p}{}^{\prime \prime }[(1-f_{l})L_{m}+c_{p,l}(T-T_{l})]\phi_{s}(T) & T_{s}<T_{p}<T_{l}\\ M_{p}{}^{\prime \prime }c_{p,l}(T-T_{p})\phi_{s}(T) & T_{p}>T_{l} \end{array}\right.$$

where, *T_p_* is the powder temperature on the liquid/gas interface which is also obtained from the laser–powder couple model. $M_{p}^{"}$ is the mass flux due to powder addition and it is given as follows:

(27) $$M_{p}^{"}=\frac{2\eta_{c}M_{p}}{\pi R_{p}{}^{2}}\exp[-2\frac{{\left(x-v_{s}t\right)}^{2}+y^{2}}{R_{p}{}^{2}}]$$

The third and fourth term of Equation (25) are the heat loss due to the thermal convection and environment radiation, respectively.

The symmetric plane is an adiabatic boundary, which indicates that heat flux could not pass through the plane. The formula of the adiabatic boundary condition is given as:

(28) $$k\nabla T\cdot n_{sp}=0$$

The bottom and side surfaces are considered as imaginary surfaces, for which the heat convection is employed as [34]:

(29) $$-k\frac{\partial T}{\partial n}=-h_{cs}(T-T_{\infty })$$

where, *h_cs_* represents the heat transfer coefficient, with an estimate value of 1250 W/(m^2^·K), based on the findings of Khandkar et al. [35].

### 3.7. Numerical Procedure

**Figure 4:**
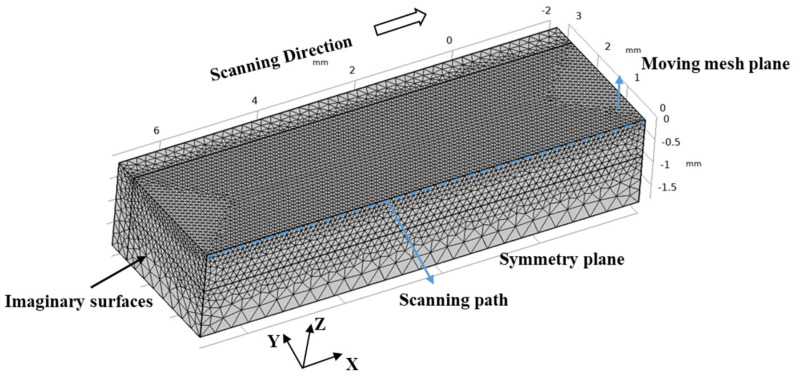
Schematic illustrations of computational domain.

The three-dimensional heat transfer model was used to conduct nonlinear transient simulation based on the commercial software COMSOL v5.4 Multiphysics. The model was built using three-dimensional Cartesian coordinate. The positive x-axis direction was consistent with the laser moving direction, the y-axis was the cross-section direction and the positive z-axis was the deposition direction. The domain with the dimension of 9 mm × 3 mm × 2 mm (x × y × z) was divided into free tetrahedron mesh, as shown in Figure 4. To calculate the temperature field, fluid dynamics and free surface moving of the molten pool accurately, finer meshes were employed near the laser scanning path. The corresponding minimum and maximum grid space were 40 μm and 100 μm, respectively, and the number of grids was about 200,000. Moreover, a time-dependent solver with adaptive time stepping was used in the model.

## 4. Results and Discussion

### 4.1. Heat Transport of Powders

**Figure 5:**
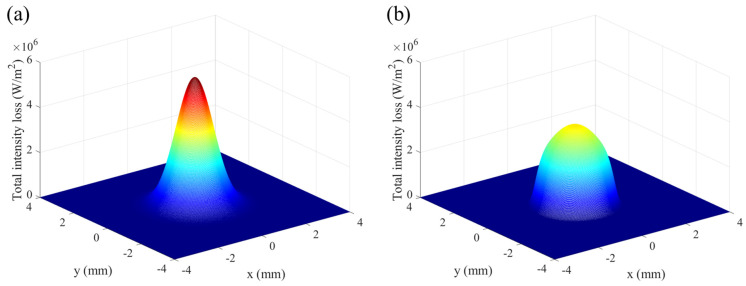
Total intensity loss after the laser reaches deposition surface under (**a**) GB, (**b**) SGB. (laser power: 2100 W; powder feeding rate: 5 g/min; laser spot: 1.6 mm; scanning speed: 10 mm/s).

As the attenuated laser beam reaches the liquid/gas interface, the total intensity loss was calculated from the sum of the intensity loss along z direction, and the formula can be given as:

(30) $$I_{loss}(r,z)=\sum_{z=z_{0}}^{z}I_{a}(r,z)[1-\exp(-Q_{ext}\pi r_{p}{}^{2}N(r,z)\Delta z)]$$

where, *I_a_* represents the attenuated laser intensity. Figure 5a,b depict the total intensity loss (TIL) when the laser reaches the deposition surface. The intensity loss has a peak value in the central region, which is attributed to the higher powder stream concentration. The peak value of the TIL for the SGB case is approximately 37.3% lower than that for the GB case and the corresponding maximum values are 3.54 × 10^6^ W/m^2^ and 5.65 × 10^6^ W/(m·K), respectively.

**Figure 6:**
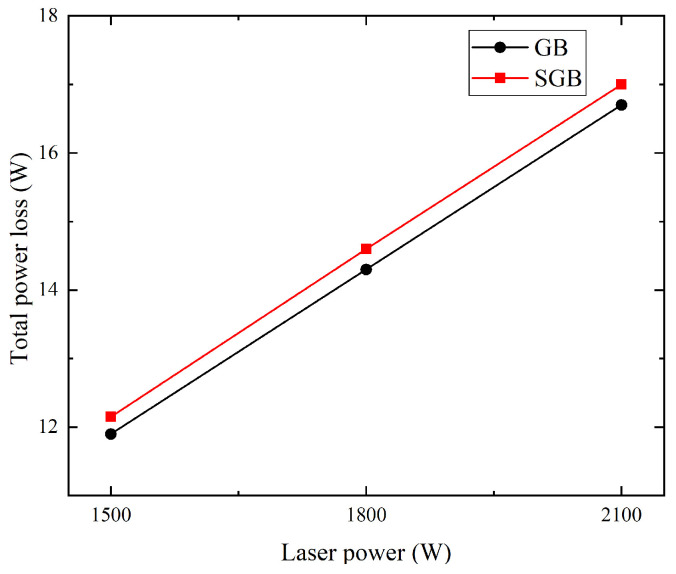
Total power loss under different laser power for the GB and SGB cases (powder feeding rate: 5 g/min; laser spot: 1.6 mm; scanning speed: 10 mm/s).

Total power loss (TPL) after laser passes through the powder stream can be calculated by integrating the TIL over the entire deposition surface and the corresponding results are shown in Figure 6. It is found that TPL grows linearly with the increase in laser power for both cases and the TPL for the SGB case is approximately 2.0% higher than that for the GB case.

**Figure 7:**
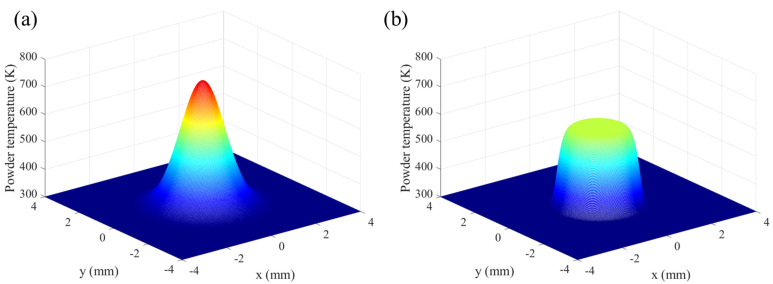
Powder temperature on the liquid/gas interface under (**a**) GB and (**b**) SGB (laser power: 1500 W; powder feeding rate: 5 g/min; laser spot: 1.6 mm; scanning speed: 10 mm/s).

The temperature of heated powder particles on the deposition surface for the GB and SGB cases are shown in Figure 7a,b, respectively. It is clearly seen that the temperature has a similar distribution with the original laser intensity and it does not exceed the melting point, which indicates that the powder has a cooling effect for the molten pool. Moreover, the peak temperature of powder is located around the laser beam center and the value is 749.4 K for the GB case while it is 581.1 K for the SGB case.

**Figure 8:**
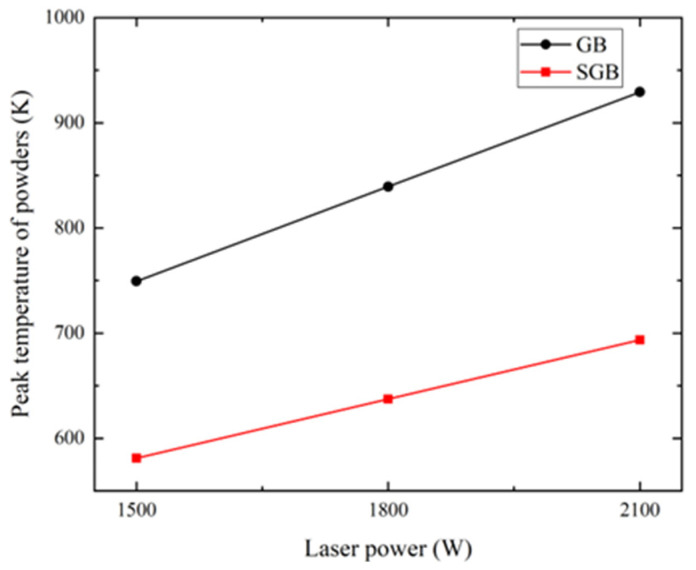
Peak temperature of powders under different laser power for the GB and SGB cases (powder feeding rate: 5 g/min; laser spot: 1.6 mm; scanning speed: 10 mm/s).

Figure 8 shows the peak temperature of powders under different laser power for the GB and SGB cases. It is found that the peak temperature grows linearly with the increase in the laser power for both the cases. However, the peak temperature is always lower for the SGB case and the rising speed is also slightly lower.

### 4.2. Temperature and Velocity Field

**Figure 9:**
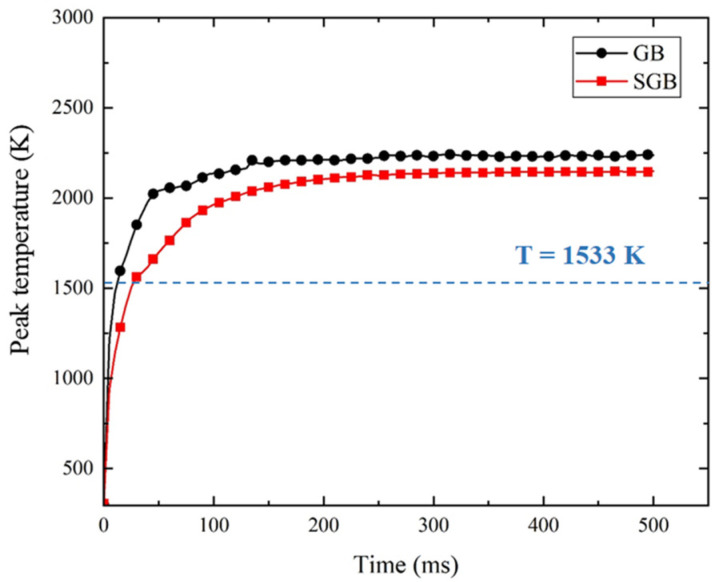
Evolution of maximum temperature in the molten pool.

To understand the evolution of temperature field in the L-DED process, the peak temperature of the molten pool under different times has been plotted in Figure 9. The peak temperature sharply rises in the initial stage before it exceeds the solidus temperature. After the molten pool forms, the temperature rises with a slower rate and gradually attains a quasi-steady state. Compared with the GB heat source, the temperature rising rate for the SGB case is slower and there is a 20 ms delay for the molten pool temperature to exceed the solidus temperature. It is found that the peak temperature has an obvious deviation after 200 ms and the calculated maximum temperature for the SGB case is 90 K lower than that for the GB case. It is indicated that the peak temperature of the system can be reduced using the SGB heat source.

**Figure 10:**
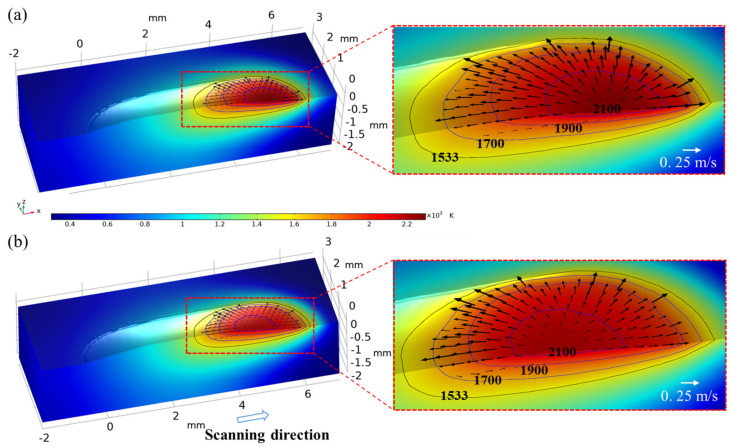
Temperature and velocity field at 500 ms for (**a**) GB and (**b**) SGB.

Figure 10 shows the temperature and velocity field of the molten pool at 500 ms after the molten pool is stabilized. Corresponding laser power (P), scanning speed (v_s_) and powder feeding rate (M_p_) are 1500 W, 10 mm/s and 5 g/min, respectively. Temperature is indicated by the contour, as shown in the figure, and the arrows represent the fluid flow direction. The black isotherm of 1533 K represents the solid temperature, and three blue isotherms are set at 1700 K, 1900 K and 2100 K, respectively. The denser isotherms indicate a greater change in temperature while sparser isotherms mean that temperature is more uniform. According to Figure 10a,b, the sparse isotherm behind the laser beam center indicates that heat accumulation occurs at the rear side while the dense isotherm at the front side indicates that the temperature is nonuniform in this region. An outward flow is spotted around the free surface of the molten pool, which is caused by the thermal capillary force that drives the liquid metal from low surface tension region to high surface tension region. Moreover, the fluid is more active for the GB case compared with that for the SGB case and the corresponding maximum fluid velocity are 0.32 m/s and 0.28 m/s, respectively.

**Figure 11:**
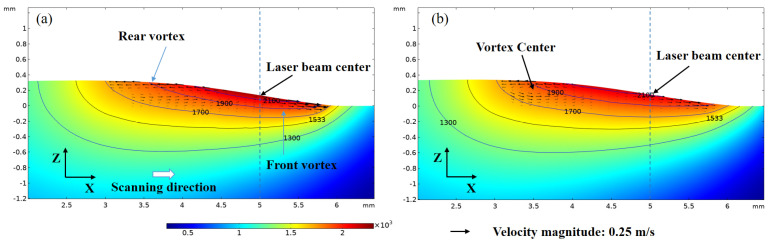
Temperature and velocity field under (**a**) GB and (**b**) SGB at xz plane(y = 0).

Figure 11 shows the temperature and velocity field at 500 ms at xz plane (y = 0). Two opposite vortices are found at the two sides of the reference line and the magnitude of the rear one is bigger. The fluid in the center of the vortex is at a standstill which means that no fluid motion occurs in this region. Moreover, the peak magnitude of vortices (∇ × **u**) for the GB case is approximately 1.5 time that for the SGB case.

**Figure 12:**
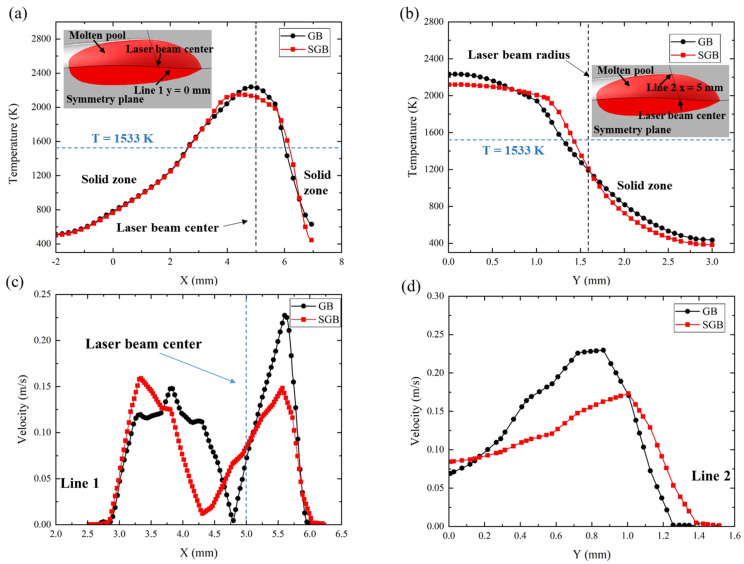
Temperature and velocity distribution along different line at 500 ms (**a**) temperature along line 1, (**b**) temperature along line 2, (**c**) velocity along line 1, (**d**) velocity along line 2.

In order to analyze the temperature and fluid distribution on the free surface, Figure 12a,b show the local temperature on the deposited surface for line 1 and line 2 at 500 ms, respectively. The deviation of the local temperature in the solid zone (temperature below 1533 K) for the GB and SGB cases is less than 3% behind the laser beam center, while a slight difference is observed in front of the center of laser beam. According to Figure 12b, temperature slightly decreases along y direction around the beam center while it decreases rapidly when it is close to the boundary. The calculated length and width under the SGB condition are 0.17 mm and 0.19 mm wider than the GB case, respectively. Figure 12c,d exhibit the fluid velocity on the free surface for the GB and SGB cases, respectively. The fluid velocity has two peak values at two sides of the laser beam center and the deviation between the rear and front side is less than 5% for the SGB case while it is larger for the GB case. Based on Figure 12d, the fluid velocity starts to increase from the laser beam center and it decreases sharply when it is close to the molten pool boundary.

To estimate the uniformity of temperature distribution of molten pool, the temperature non-uniformity $\delta_{T}$ is calculated. The formula of temperature non-uniformity is as follows [10]:

(31) $$\delta_{T}=\frac{\sum_{i}V_{i}|\frac{T_{i}-T_{ave}}{T_{ave}}|}{\sum_{i}V_{i}}$$

where, *i* deontes the *i*th cell and *V_i_* represents the cell volume. *T_ave_* is the average temperature of the molten pool and is calculated using volume averaging method.

It should be noted that the temperature non-uniformity is calculated inside the molten pool whose temperature is above the solid temperature. The greater uniform temperature inside the molten pool leads to the smaller value of *δ_T_*. The calculated temperature non-uniformity is 0.073 and 0.072 for the SGB and GB cases, respectively. Even though the distribution of energy input is quite different, temperature field inside the molten pool for these two cases is almost the same. Additionally, the calculated temperature non-uniformity is used to help explain the fluid motion in next section.

**Figure 13:**
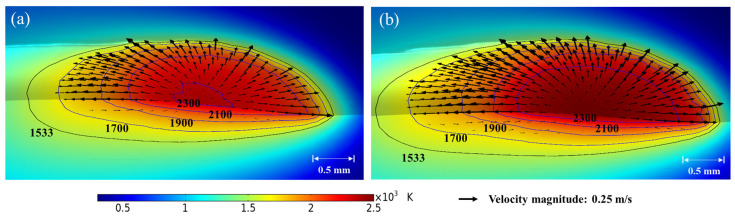
Temperature and velocity field under different laser power for the SGB case at 500 ms (**a**) 1800 W, (**b**) 2100 W.

A parametric study has been performed to investigate the influence of the laser power on the evolution of temperature and velocity in the molten pool. Two different cases under the SGB energy input have been analyzed here and the input power is 1800 W, and 2100 W, respectively. Figure 13a,b shows the temperature and velocity field under different laser power after the molten pool is stabilized, respectively. It is found that the increase in molten pool size is due to the increase in heat input. Moreover, a more melting volume above 2300 K is observed as the laser power increase.

Based on the simulated results, the melting volume, peak temperature, peak velocity and temperature non-uniformity have been presented in Table 3. It is clearly seen that the melting volume, peak temperature and peak velocity are larger due to the increase in laser power. The increase in fluid velocity can be attributed to less uniform temperature, which is consistent with the calculated temperature non-uniformity.

### 4.3. Dimensionless Analysis

Thermal conduction and convection are two mechanism of heat transfer in the L-DED process. To find which one is dominate, a non-dimensional Peclet number (*Pe*) is used to evaluate the relative importance and it is defined as the ratio of momentum and energy diffusion term. The related formula is as follows:

(32) $$Pe=\frac{\rho C_{p}\bar{U}L_{c}}{k}$$

where, $\bar{U}$ is the average fluid velocity within the molten pool, and it is calculated by using volume average method and the formula is expressed as:

(33) $$\bar{U}=\frac{\sum_{i}V_{i}u_{i}}{\sum_{i}V_{i}}$$

*L_c_* is the equivalent molten pool radius which is calculated by converting the molten pool volume to the equivalent hemisphere and the formula is given as:

(34) $$r_{a}=\sqrt[{3}]{\frac{3V_{m}}{2\pi }}$$

**Figure 14:**
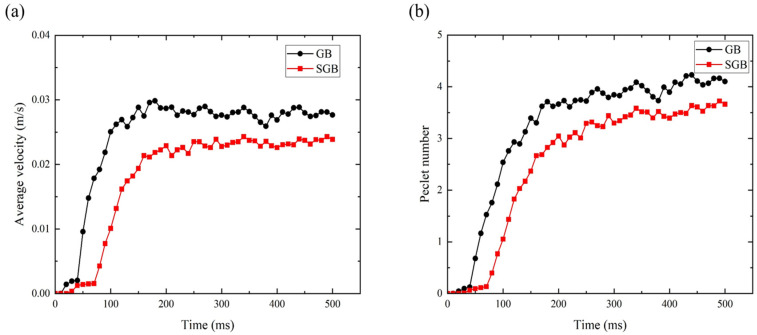
Average liquid velocity (**a**) and the Pe number (**b**) against time for the GB and SGB cases.

The average liquid velocity within the molten pool and calculated Peclet number (*Pe*) have been plotted in Figure 14. The fluid velocity is zero in the initial stage because the material does not reach the melting point and it is kept at a certain level after the system reaches a stabilized condition. Moreover, the average liquid velocity under the GB condition is approximately 16% higher than that for the SGB case, which means that the entire fluid motion is more active for the GB case. Based on Figure 14b, the Pe number has the same tendency with the average velocity. Except for the beginning stage, the value of Pe exceeds the unit, underlining that thermal convection dominated the mechanism of heat transfer. The calculated melting volume for the SGB case is 1.32 × 10^−9^ m^3^, while it is 1.19 × 10^−9^ m^3^ for the GB case, indicating an almost 10% bigger melt volume for the SGB case, which results in a larger apparent molten pool radius. Although the characteristic length under the SGB condition is about 3.4% wider than the GB case, the calculated Pe number under the GB condition is larger (Figure 14b), which is attributed to the larger magnitude of fluid velocity. It is noted that the Pe number for the GB case is approximately 12% larger than that for the SGB case at 500 ms, meaning that the thermal convection has a larger influence on heat transfer compared to thermal conduction.

Thermal capillary force results in the fluid flow on the free surface is known as Marangoni–Benard convection. Buoyancy results in the fluid flow in the entire molten pool is known as Rayleigh–Benard convection.

Marangoni number (*Ma*) is defined as the ratio of surface shear stress and viscous shear stress which evaluates the Marangoni shear stress of the metal fluid. It is given as [6]:

(35) $$Ma=\frac{\rho \Delta T_{\max}L_{c}|\gamma |}{\mu \alpha }$$

where, $\Delta T_{\max}$ represents the deviation between the melting point and peak temperature inside the molten pool. *α* is the thermal diffusivity.

Rayleigh number (*Ra*) is used to express the relative strength of buoyancy and viscous force within the molten pool and it is given as [36]:

(36) $$Ra=\frac{\rho \beta g\Delta T_{\max}L_{c}{}^{3}}{\mu \alpha }$$

**Figure 15:**
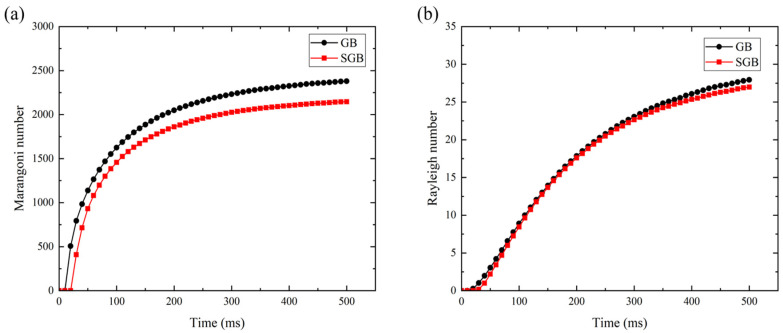
Plot of Ma number (**a**) and Ra number (**b**) against time for the GB and SGB cases.

Figure 15a,b show the variation of Marangoni and Rayleigh number with time within the molten pool under the GB and SGB conditions, respectively. It should be noticed that the Marangoni number is much larger than the unit, meaning that the thermal capillary force is much larger than the viscous force for both cases. From Figure 15a, it is found that the Marangoni number under SGB condition is always lower than that under the GB condition which is caused by the uniform energy. Moreover, thermal capillary for the GB case has more significant influence on the molten pool dynamics, which is caused by the non-uniform heat input on the deposited surface. From Figure 15, it is found that the Ra number is approximately three orders of magnitude smaller than the Ma number, which is mainly due to the small characteristic length (of the order of 10^−3^ m) of the molten pool. In this respect, Marangoni effect dominates the fluid flow pattern in the L-DED process.

It is worth mentioning that the Prandtl number (Pr = μc_p_/k), which is closely related to the location of vortices, has a significant influence on the fluid dynamics and further on the appearance of the molten pool [37]. Pr number is used to describe the boundary layer during the molten pool evolution. The calculated Pr number (in this case Pr = 0.14) is less than unit, which indicates that the momentum dissipation is the primary mechanism.

The Fourier number (*Fo*) is a dimensionless number which is related to the two opposite mechanisms of heat dissipation and heat accumulation. A high Fourier number indicates that the heat within the molten pool can be dissipated more easily and the formula can be expressed as [6]:

(37) $$Fo=\frac{\alpha }{v_{s}L_{c}}$$

**Figure 16:**
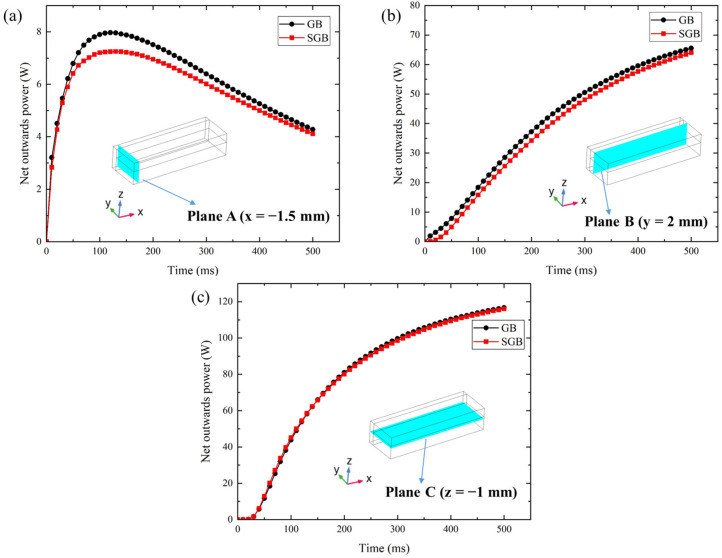
Net output power calculated on a plane versus time (**a**) x = −1.5 mm, (**b**) y = 2 mm, (**c**) z = −1 mm.

For the GB and SGB cases, the calculated Fourier numbers are 0.544 and 0.526 at 500 ms, respectively. The former is approximately 3.4% larger than the latter, which is attributed to the bigger melting volume. Thus, less Fo number indicates that the molten pool for the SGB case has a weaker heat dissipation capability, and on the other hand, more heat is accumulated inside the molten pool rather than dissipated to the no-melting region. To explain this process, the thermal transfer towards the no-melt region has been calculated on three selected planes, as shown in Figure 16. Here, the net output power (*NOP*) is defined as the integration of heat flux through the selective plane, the formula can be expressed as:

(38) $$NOP=\sum_{i}A_{i}q_{i}$$

where, *A_i_* represents the area of *i*th cell in the selective plane and *q_i_* denotes the heat flux through the selective plane. Based on Figure 16, although the predicted NOP has the same tendency in the two cases at three planes, an obvious difference can be discovered at plane A and B while its difference is less than 3% at plane C. Hence, the heat dissipation capability is weaker for the SGB case due to its relatively lower fluid velocity, as shown in Figure 12 and Figure 14. It is also found that fluid flow will significantly influence the heat transport between the molten pool and the no-melt region in LPBF [38].

For further analysis of the thermal-fluid transport inside the molten pool, the relevant non-dimensional numbers have been calculated and presented in Table 4. It can be found that Pe number is directly related to the heat input and an increase in laser power will strength heat convection. Moreover, an increase in Ma and Ra number indicates that both Marangoni and buoyance effect are strengthened by higher laser power. In addition, because the ratio of Ma number and Ra number is on the order of 100, Marangoni effect is the main physical mechanism dominating the driving forces for liquid flow. From Table 4, it is evident that the Fo number decreases as the heat input increases, which indicates that the heat dissipation capability is weaker when the laser power rises.

### 4.4. Solidification Characteristics

Grain morphology of metals during the L-DED process depends on solidification parameters. Temperature gradient (***G***) is a solidification parameter and its direction is normal to the solidification front. The model in this paper is based on the Cartesian coordinate system, thus ***G*** is expressed as:

(39) $$G=(\frac{\partial T}{\partial x},\frac{\partial T}{\partial y},\frac{\partial T}{\partial z})$$

Growth velocity *R* is another solidification parameter which is normal to the liquid/gas interface and its magnitude can be expressed as:

(40) $$R=v_{s}\cdot \frac{\partial T}{\partial x}/G$$

**Figure 17:**
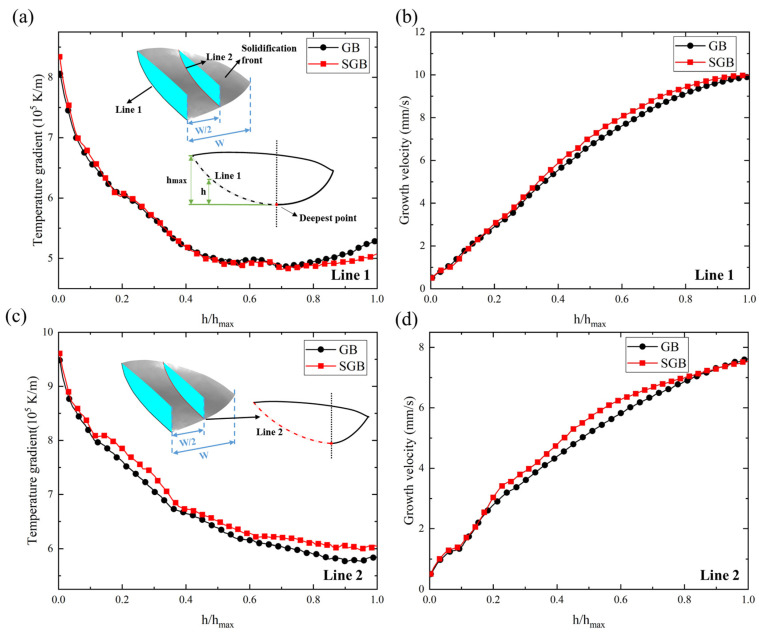
Temperature gradient and growth velocity under the laser power of 1500 W for the GB and SGB cases along different path: (**a**) temperature gradient for line 1, (**b**) growth velocity for line 1, (**c**) temperature gradient for line 2 and (**d**) growth velocity for line 2.

The solidification parameter *G* × *R* is related to the grain size, representing the instantaneous cooling rate on the solidification front. *G*/*R* is another solidification parameter which has important relevance to the microstructural morphology [39]. *G* and *R* along line 1 for the GB and SGB cases are shown in Figure 17a,b, respectively. It is found that *G* first decreases then a slight increase is observed around the top surface for both the cases. The maximal magnitude of *G* is located at the bottom region and the corresponding value is 8.1 × 10^5^ K/m and 8.4 × 10^5^ K/m, respectively. The slight increase in *G* at the top region is mainly due to the thermal exchange, including convection and radiation with the external environment. Growth velocity of the solidification front increases from 0.51 mm/s and 0.50 mm/s at the bottom region to 9.8 mm/s and 10 mm/s at the top region for the GB and SGB cases, respectively. The top region in the center has a growth velocity which is almost the same with the scanning speed, meaning that the local growth direction is consistent with the laser moving direction. However, *R* at the bottom region is approximately 3% of that at the top for both cases. *G* and *R* along line 2 are plotted in Figure 17c,d, respectively. The variation tendency of *G* and *R* along line 2 is similar to those along line 1. However, temperature gradient along line 2 is always higher for the SGB case, and the growth velocity in the central region for the SGB case is larger than that for the GB case, while it is almost the same on two sides.

**Figure 18:**
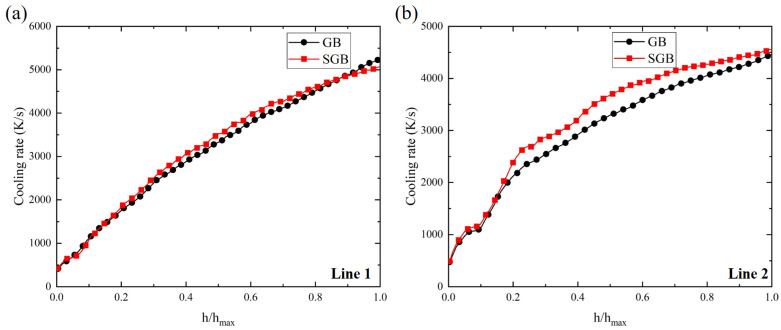
Cooling rate under the laser power of 1500 W for GB and SGB cases along different path: (**a**) line 1 and (**b**) line 2.

Figure 18a,b show the change in *G* × *R* at the line 1 and line 2, respectively, with the relative height of the solidification front and the corresponding laser power is 1500 W. *G* × *R* increases from 412 K/s to 5264 K/s from the bottom region to the top region for the GB case while it increases from 420 K/s to 5069 K/s for the SGB case. From Figure 18a, *G* × *R* is lower at the top region for the SGB case, while it is larger in the middle region. However, the relative difference is less than 5%. According to Figure 18b, *G* × *R* increases with the increase in relative height and it reaches 4455 K/s and 4561 K/s for the GB and SGB cases, respectively. Moreover, it can be found that *G* × *R* along line 2 is higher for the SGB case, which indicates that the grain size could be smaller for the SGB case. The higher magnitude of *G* × *R* is attributed to the higher temperature gradient and growth velocity according to Figure 17c,d.

**Figure 19:**
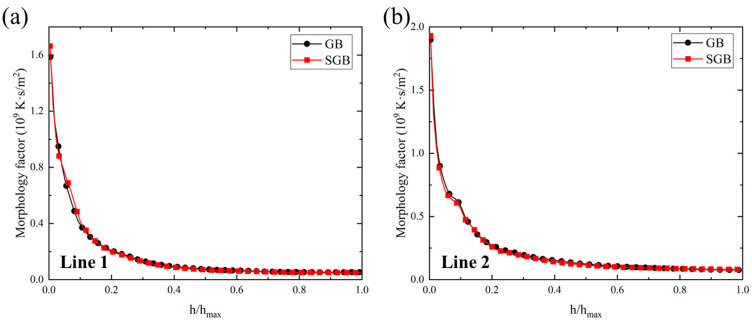
(**a**) Morphology factor under the laser power of 1500 W for GB and SGB cases along different path: (**a**) line 1 and (**b**) line 2.

Figure 19a,b show the morphology factor (*G*/*R*) along the line 1 and line 2, respectively. It is found that with the increase in relative height, the morphology factor rapidly first decreases, after that, it decreases with a slower speed until reaching the molten pool surface. Thus, it can be predicted that columnar growth appears more likely at the bottom region for both the cases. The relative error in this region for the two lines is less than 5%. Comparing the morphology factor along the different paths, it is found that *G*/*R* is larger along line 2, which is mainly caused by the higher magnitude of *G* and *R* according to Figure 17. Based on Figure 19a, the maximum value of the solidification parameter *G*/*R* is located at the bottom region and the corresponding values are 1.59 × 10^9^ K·s/m^2^ and 1.68 × 10^9^ K·s/m^2^ for the GB and SGB cases, respectively.

**Figure 20:**
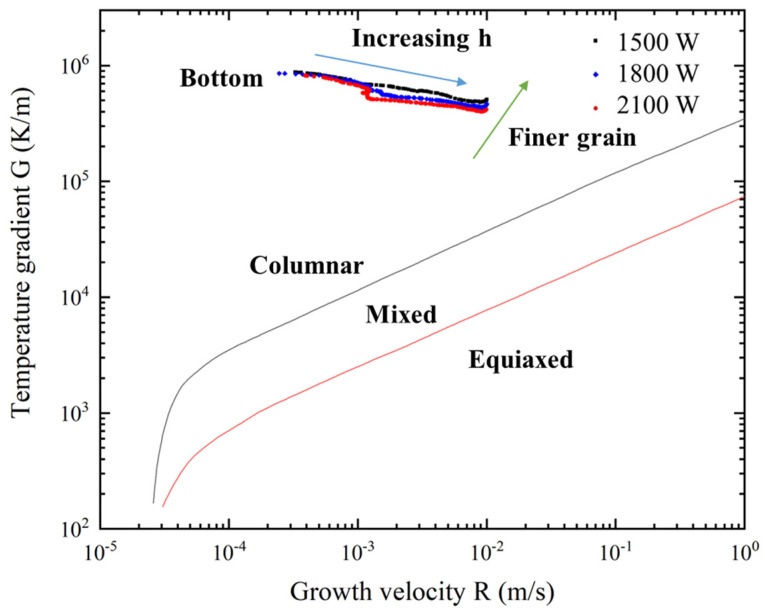
Predicated temperature gradient and growth velocity along line 1 plotted on reference solidification map [40] under different laser power.

Figure 20 shows the *G* and *R* along line 1 plotted on a reference solidification map, indicating the influence of laser power on grain size and morphology [40]. The arrows indicating “finer grain” correspond to the direction of increasing cooling rate, which indicates that the grains can be refined by lowering the heat input. It is evident that grain morphology along line 1 under different laser power is columnar especially for the bottom region, which is mainly caused by the smaller *R* and higher *G*.

To further analyze the solidification parameters under different cases, the average solidification parameters are calculated using the area-weighted average method and its formula is as follows:

(41) $$\bar{f}=\frac{\sum_{i}A_{i}f_{i}}{\sum_{i}A_{i}}$$

where, $A_{i}$ represents the area of *i*th cell of the solidification front and *f_i_* denotes the solidification parameter. In this equation, *f_i_* should be replaced by *G*, *R*, *G* × *R* and G/R.

The calculated average solidification parameters are presented in Table 5. It is found that the average cooling rate is 11.1% higher for the SGB case under the laser power of 1500 W, which means that the grains could be finer for the SGB case in the L-DED process. Moreover, as the laser power increases from 1500 W to 2100 W, the average cooling rate decreases from 2.40 × 10^3^ K/s to 1.71 × 10^3^ K/s, which indicates that the average grains size in the clad layer would become larger with the increase in the energy input.

Inconel 718 is a typical alloy that has large solidification ranges due to its several alloying elements [41]. During the solidification process, the undercooled region is formed and several crystal structures, such as stable planar crystals, unstable cellular crystals and dendrites, may be produced in this region. [39]. Research by previous scholars [15,16] have revealed that the solidification morphology of Inconel 718 during rapid solidification processes might be either cellular or dendritic, which depends on the experimental parameters. The transformation of columnar to equiaxed transition (CET) is dependent on the constitutional supercooling (CS) [42], which is usually described by the solidification parameter *G*/*R*. Lower magnitude of *G*/*R* results in more CS in the solidification front, which brings more possibility for the equiaxed grain growth. As the undercooling in the solidification front exceeds the required subcooling of the nucleation, equiaxed crystals can precede columnar nucleate.

In considering multi-component solute systems of Inconel 718 alloy, the constitutional tip undercooling is related to the solute concentration [43].

(42) $$\Delta T_{c}=\sum_{i=1}^{n}m_{i}(C_{0,i}-C_{l,i}^{*})$$

where, *m_i_* is the slope of the liquidus. Additionally,$C_{0,i}$ is the nominal concentration of the alloys and $C_{l,i}^{*}$ denotes the concentration at the tip in the liquid. The thermodynamics and kinetics of the CET are simplified by using an empirical relation, as presented by Gäumann et al. [43]:

(43) $$\frac{G^{n}}{R}=a{\left[\sqrt[{3}]{\frac{-4\pi N_{0}}{3\ln(1-\phi )}}\frac{1}{n+1}\right]}^{n}$$

where, *a* and *b* are constants related to the alloy, *N*_0_ denotes the nucleation density, and *ϕ* is the volume fraction of equiaxed grains which is also called stray grains (SGs). According to Hunt’s research [42], the system can be considered fully columnar growth when the volume fraction of the SGs is below 0.66%. While the volume fraction exceeds 49%, the system is considered as fully equiaxed growth. Between these two regions, there is mixed columnar/equiaxed grain growth. In this respect, the pattern of grain growth can be predicted by calculating *G* and *R*.

The fraction of the SGs can be calculated by rearranging Equation (43).

(44) $$\phi =1-\exp\left\{\frac{-4\pi N_{0}}{3}{\left(\frac{1}{\left(n+1)(G^{n}/aR\right)}\right)}^{3}\right\}$$

It is observed that the fraction of the SGs depends on the material and solidification parameters. The constants associated with alloys and nucleation density are using the parameters reported by Gäumann et al. [43] for a similar nickel-base superalloy while the solidification parameters are calculated in the 3D numerical model. The average volume fraction of the SGs is calculated using the same method as the average solidification parameters and it is given as [44]:

(45) $$\bar{\phi }=\frac{\sum_{i}A_{i}\phi_{i}}{\sum_{i}A_{i}}$$

**Figure 21:**
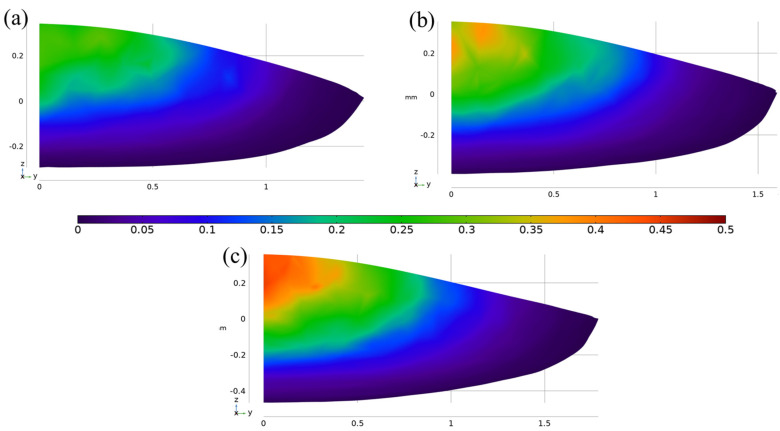
Predicted volume fraction of the SGs on the solidification plane for the SGB case under different laser powers (**a**) 1500 W (**b**) 1800 W (**c**) 2100 W.

Three predicted volume fraction maps of the SGs under different laser power are presented in Figure 21. It is shown the formation possibility of the SGs and the value of *ϕ* in the central-top region is the maximum for three different cases. Moreover, the predicted maximal volume fraction of the SGs reaches 0.3 for the SGB1500 case, while it reaches 0.47 the SGB2100 case. Thus, it can be predicted that *ϕ* could increase as the heat input increases.

**Figure 22:**
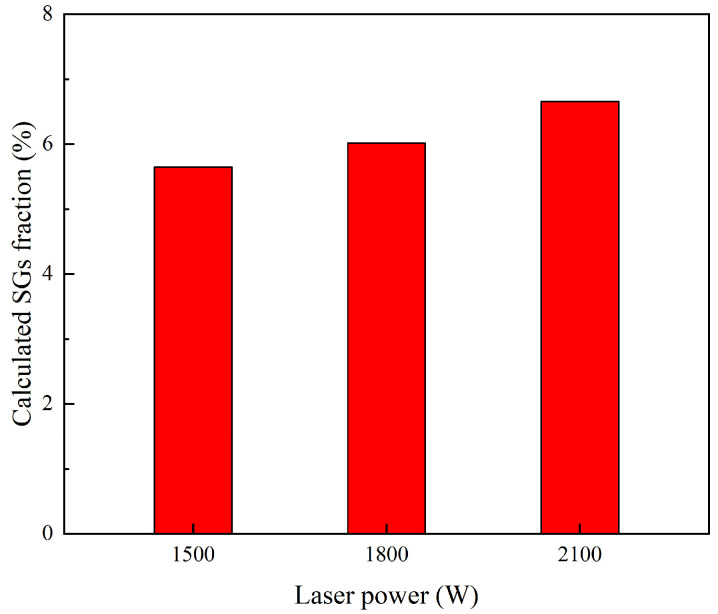
Predicted average volume fraction of the SGs under different laser powers.

The calculated average volume fraction of the SGs under different laser powers is plotted in Figure 22. It is found that the predicted average fraction of the SGs is 5.6% with a laser power of 1500 W, while it increases to 6.6% with a laser power of 2100 W. Thus, low heat input conditions are helpful to avoid the formation of the SGs and promote directional grain growth.

### 4.5. Validation of the Model

**Figure 23:**
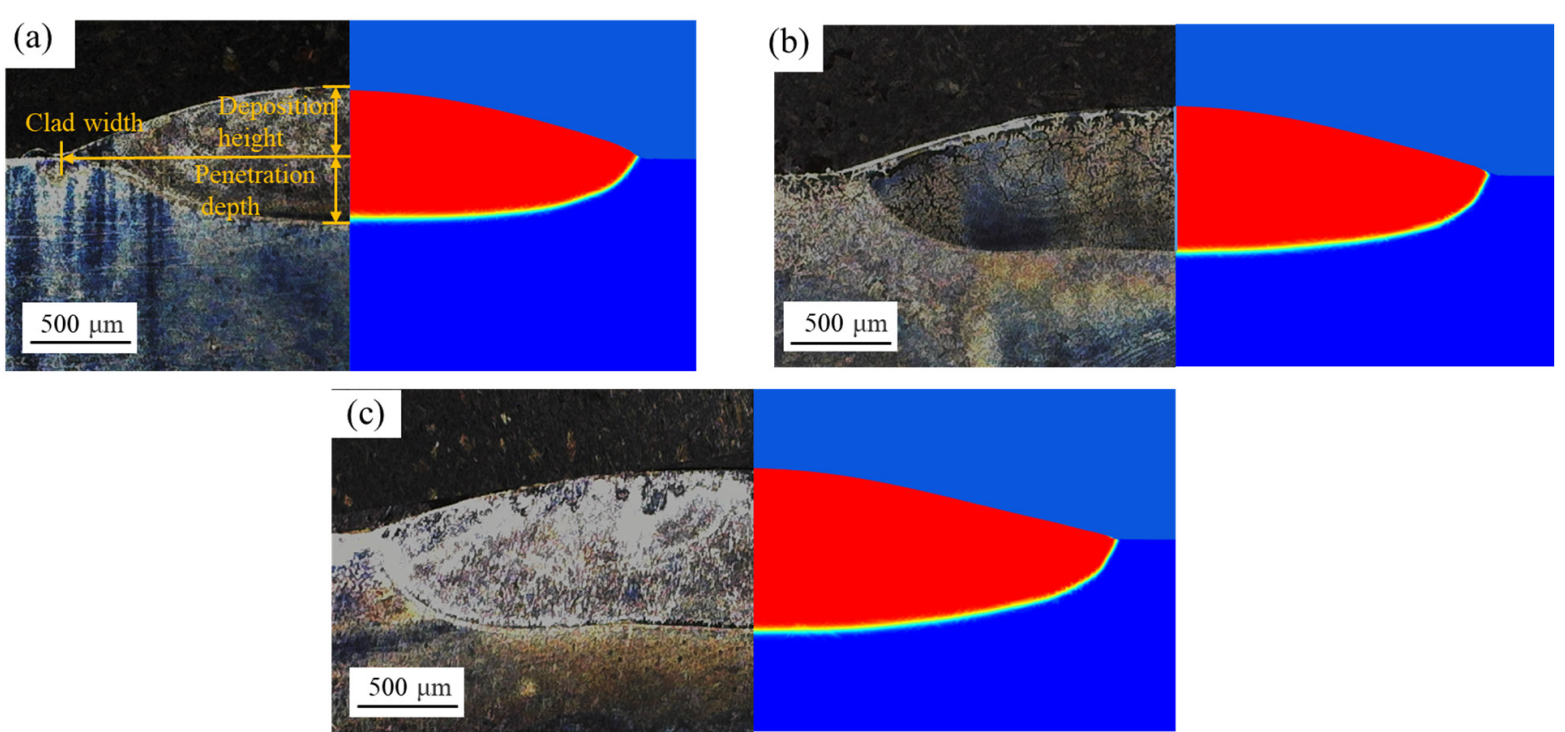
Experimental and computed cross-sections of the clad layer for the SGB case at different laser powers: (**a**) 1500 W, (**b**) 1800 W and (**c**) 2100 W.

The cross-sectional morphology of the clad layer, derived from experiments and computation, is plotted on Figure 23. The results show that predicted geometry by numerical simulation matches well with the experiment results.

Table 6 shows the comparison of geometric parameters for cross-section of the clad layer between the experimental and simulated results. It is found that the width and depth of the cladding layer increase as the heat input increases. However, the deposition height is almost the same for three cases. This phenomenon is due to two opposite factors, the flow of metal liquid and the “catch” efficiency of the liquid metal. On one hand, increasing the heat input leads to a larger molten pool size which could catch more powder particles, thus the deposition height is larger. On the other hand, the flow of metal liquid is also strengthened with the increase in heat input, causing liquid metal from the center to flow towards the edge.

The results show that the prediction error of the clad width is less than 5.4%, penetration depth error is less than 6.3% and deposition height error is less than 7.0%, which means that the prediction of the numerical simulation is good. From the validations, it is observed that the computed results have some differences with the experimental results, which can be attributed to many factors, such as neglect of shielding gas and vaporization.

## 5. Conclusions

The thermal-fluid transfer behavior and solidification characteristics under two types of laser beam, the GB and SGB, are analyzed utilizing an improved three-dimensional numerical model. Based on the numerical simulation, the following specific conclusions can be drawn:

1. The total attenuation of laser intensity has a Gaussian-like shape for both cases. During the laser–powder interaction, the temperature of the heated powder particles is lower than the melting point of the metal. Moreover, the particle temperature on the liquid/gas interface has a similar distribution with its original laser intensity.
2. The laser beam profile has a significant influence on the temperature field and fluid flow in the molten pool. Compared with the GB case, the peak temperature for the SGB case is approximately 90 K lower, and the average liquid velocity within the molten pool is about 14% lower.
3. The main heat dissipation mechanism in the molten pool is convection rather than conduction, because Pe number is much larger than the unit. The Peclet number for the GB case is approximately 12% larger than that for the SGB case, meaning that the fluid flow has a greater effect on heat transfer compared with thermal conduction. Moreover, thermal capillary force is the main driving force for the GB and SGB cases.
4. The average temperature gradient and cooling rate are both higher for the SGB case which indicates that the grains could be finer for the SGB case in the L-DED process. Additionally, the higher magnitude of morphology factor for the SGB case means that it has more possibility for columnar growth.

## Figures and Tables

**Table 1:** Processing parameters used in L-DED experiments.

Processing Parameters	Laser Spot Radius (mm)	Powder Feeding Rate (g/min)	Laser Power (W)	Scanning Speed (mm/s)
value	1.6	5	1500–2100	10

**Table 2:** Material properties of Inconel 718 applied in calculation [33].

Property	Value	Unit
Density of solid, *ρ_s_*	7676	kg/m^3^
Density of liquid, *ρ_l_*	7400	kg/m^3^
Liquid viscosity, *μ*	0.006	Pa·s
Solid temperature, *T_s_*	1533	K
Liquid temperature, *T_l_*	1609	K
Latent heat of fusion, *L_f_*	2.09 × 10^5^	J/kg
Thermal conductivity, *k*	0.5603 + 0.0294T − 7.0 × 10^−6^ T^2^	W/(m·K)
Specific heat of solid, *C_p,s_*	625	J/(kg·K)
Specific heat of liquid, *C_p,l_*	725	J/(kg·K)
Surface tension coefficient, *γ*	−0.00011	N/(m·K)
Thermal expansion coefficient, *β*	1.63 × 10^−5^	1/K
Stefan-Boltzmann constant, *σ*	5.67 × 10^−8^	W/(m^2^·K^4^)
Equivalent emissivity, *ε*	0.5	-

**Table 3:** Melting volume, peak temperature, peak velocity and temperature non-uniformity under different laser power.

Case	Melting Volume (m^30^)	Peak Temperature (K)	Peak Velocity (m/s)	Temperature Non-Uniformity (-)
SGB1500	1.32 × 10^−9^	2149	0.28	7.29 × 10^−2^
SGB1800	2.05 × 10^−9^	2322	0.34	8.56 × 10^−2^
SGB2100	2.86 × 10^−9^	2481	0.39	9.60 × 10^−2^

**Table 4:** Dimensionless numbers under different laser power.

Case	$\bar{U}$ (cm/s)	Pe	Ra	Ma	Fo
SGB1500	2.4	31.8	26.9	2146.8	0.526
SGB1800	3.0	46.2	53.6	3184.9	0.454
SGB2100	3.4	58.8	89.8	4271.8	0.407

**Table 5:** Calculated average of the solidification parameters.

Case	Temperature Gradient (K/m)	Growth Velocity (m/s)	Cooling Rate (K/s)	Morphology Factor (K·s/m^2^)
GB1500	7.72 × 10^5^	3.16 × 10^−3^	2.16 × 10^3^	6.16 × 10^8^
SGB1500	7.98 × 10^5^	3.36 × 10^−3^	2.40 × 10^3^	6.60 × 10^8^
SGB1800	7.76 × 10^5^	2.93 × 10^−3^	1.99 × 10^3^	1.66 × 10^9^
SGB2100	7.38 × 10^5^	2.69 × 10^−3^	1.71 × 10^3^	1.72 × 10^9^

**Table 6:** Experimental and computed geometric parameters.

Case	Clad Width (mm)			Penetration Depth (mm)			Deposition Height (mm)		
	Exp.	Sim.	Error (%)	Exp.	Sim.	Error (%)	Exp.	Sim.	Error (%)
SGB1500	1.46	1.45	0.6%	0.32	0.30	6.3%	0.35	0.34	2.3%
SGB1800	1.57	1.62	3.2%	0.39	0.40	1.8%	0.34	0.36	4.7%
SGB2100	1.88	1.78	5.4%	0.46	0.47	2.0%	0.34	0.36	7.0%

## Data Availability

Not applicable.
